# Predictive Value of CT Brain Perfusion Studies in Acute Ischemic Infarct Taking MRI Stroke Protocol As Gold Standard

**DOI:** 10.7759/cureus.16501

**Published:** 2021-07-20

**Authors:** Hafeez-ur-Rehman Junejo, Shazia Yusuf, Romasa Zeb, Uswa Zeb, Ahmed A Zeb, Aamena Ali

**Affiliations:** 1 Radiology, Bilawal Medical College, Jamshoro, PAK; 2 Diagnostic Radiology, Capital Hospital, Islamabad, PAK; 3 House Officer Medicine, Capital Hospital, Islamabad, PAK; 4 Medicine, Capital Hospital, Islamabad, PAK; 5 Medicine, Army Medical College, Rawalpindi, PAK

**Keywords:** acute ischemic infarct, stroke, ct perfusion, mri, dwi

## Abstract

Background

Acute ischemic stroke is the leading cause of serious chronic disability worldwide. Imaging plays a key role in early diagnosis and intervention, thus reducing mortality and morbidity related to ischemic stroke. Computed tomography (CT) perfusion study is a valuable imaging tool for the assessment of acute infarction. The objective of this study was to determine the predictive value of CT perfusion in diagnosing acute ischemic infarction taking Magnetic Resonance Imaging (MRI) stroke protocol (including Diffusion Weighted Imaging (DWI)) as a gold standard.

Methods

The cross-sectional validation study was conducted at a teaching hospital in Islamabad from June 2019 to December 2019. The study comprised a total of 125 patients of either gender with suspected acute ischemic stroke. The patients were scanned for CT perfusion and MRI stroke protocol on the same day. Scans were reported separately for the detection of acute ischemic infarction by the same consultant radiologist. The predictive value of CT perfusion was calculated accordingly.

Results

Of the 125 patients, 58% were male and 42% were female. The age of selected patients ranged between 38 to 70 years with a mean age of 56.12 ± 9.69 years. Acute ischemic infarction was detected in 86 (69%) patients by CT perfusion study and in 120 (96%) patients by MRI stroke protocol. The positive predicted value of CT perfusion for the detection of acute infarction was calculated as 98.83 and the negative predicted value was 10.25.

Conclusion

CT perfusion study provides adequate sensitivity and specificity with good predictive value in the detection of acute ischemic infarct in stroke patients. This widely available and time-effective modality aids in the triage of patients for immediate endovascular intervention leading to maximal neurological benefit and improving outcomes.

## Introduction

Stroke is one of the common causes of morbidity and mortality throughout the world. It is the second major cause of death and the third major cause of disability worldwide [1]. The annual incidence of stroke in Pakistan is about 250/100,000 corresponding to 500,000 cases of stroke yearly [2,3]. An ischemic stroke is any thromboembolic or other event resulting in compromised blood flow to brain tissue [4]. The irreversibly damaged area of the cortex is the ischemic core and peripheral reversible zone of ischemia - “ischemic penumbra” [5]. The goal of the treatment is to restore flow to ischemic penumbra - the salvageable tissue around the infarct [6].

Tissue plasminogen activator (tPA) is a standard treatment in ischemic stroke patients [7]. Studies suggest the use of tPA along with timely endovascular reperfusion therapy can decrease disability to a greater extent and ensure normal or near-normal recovery [8]. The selection of candidates for endovascular reperfusion depends upon the determination of core to penumbra ratio [9]. The size of the penumbra is a predictor of neurologic outcome after reperfusion therapy. On the one hand, a large area of infarction with a small area of penumbra is considered a contraindication for endovascular reperfusion due to the risk of hemorrhage and low likelihood of a good outcome. On the other hand, the patients with larger penumbra can greatly benefit from the immediate therapy as it would lead to revascularization of the salvageable area and correspondingly decreased disability later in life [10,11].

For the diagnosis of acute brain ischemic changes, MRI stroke protocol is a more sensitive and more accepted modality. MRI remains the gold standard for determining the infarct core [12]. However, MRI has a number of limitations and cannot be used in certain circumstances, i.e., irritable/restless patients and the patients having a metallic prosthesis. Another imaging modality with similar results in hyperacute stroke setting is CT perfusion. CT perfusion study predicts the infarct core rapidly and accurately with the ability to differentiate between irreversible infarcted core and reversible surrounding ischemic tissue - the penumbra [13].

Timely diagnosis is of primary importance in proceeding for intravenous thrombolysis and mechanical thrombectomy in hyperacute stroke patients [14]. CT perfusion studies are more suitable in the emergency setting to diagnose and select acute stroke patients that can benefit from endovascular reperfusion and thrombolytic therapy; due to its wide availability and considerably less time requirement [15,16]. This technique can be adapted in emergency facilities for acute stroke setting to facilitate diagnosis, aiding in establishing management plans of patients thus overall improving outcomes and optimizing expenses.

The purpose of this study was to determine the predictive value of CT perfusion in diagnosing acute ischemic infarct taking MRI stroke protocol (including Diffusion Weighted Imaging(DWI)) as a gold standard.

## Materials and methods

After approval from the institutional ethical review board, the present cross-sectional validation study was conducted at the Radiology Department of a teaching healthcare facility in Islamabad from June 2019 to December 2019. The sample size was calculated by using the WHO sample size calculator taking sensitivity of CT perfusion 69.9%, specificity as 87.4%, and prevalence of acute ischemic stroke 67 %, the estimated sample size was 125.

The age of patients ranged between 38 - 70 years, male or female gender, suspected of acute ischemic stroke - those with symptoms of focal neurological deficit lasting for ≥ 24 hours were included in the study. Those patients diagnosed with hemorrhagic or mixed (hemorrhagic + ischemic) stroke and those diagnosed with venous infarcts were excluded from the study. After informed consent, 125 patients with suspected acute ischemic stroke were included in the study using non-probability consecutive sampling. All the patients were entitled to medical treatment as the hospital belongs to a department of the federal government, and the employees and their families were entitled to free health services in the hospital. Hence the services of CT and MRI were free for these patients. The patients were scanned for CT perfusion and MRI stroke protocol on the same day.

A 64‑slice spiral CT scanner machine (Toshiba, Minato City, Tokyo, Japan) was used to first perform non-contrast CT (NCCT) brain to exclude hemorrhage (hyperdense area) and large areas of clearly infarcted tissue (hypodense area); then one slice with suspected acute infarction was selected for CT perfusion study by the consultant radiologist with experience of 7+ years. The scanning layer thickness was 8 mm, matrix 512x512, tube voltage 120 kV, tube current 70 mA, interval time: 1 sec; scanning time: 53 sec, a total of 80 layers, and scanning range was 80 mm. 50 ml of 370 mg/ml concentration of non‑ionic iodine contrast agent (Inj. Lopromide) was injected through the right antecubital vein at a flow rate of 5.0 to 6.0 ml/sec, followed by 50 ml saline at 5.0 to 6.0 ml/sec and scanned at the same time (delay after injection was 7 sec). The scan was performed, and the CT image data was transmitted to the workstation, and Vitrea software (Toshiba, Minato City, Tokyo, Japan) was used for analysis. CT perfusion parameters, namely Cerebral Blood Flow (CBF), Cerebral Blood Volume (CBV), and Mean Transit Time (MTT), were measured. Acute infarction on perfusion study appeared as the area of the brain with prolonged relative MTT (> 145%), markedly decreased CBF (< 25 ml x 100 g-1 x min-1), and markedly reduced CBV (< 2 ml x 100 g-1) in comparison to the contralateral normal side.

MRI study was performed on a 1.5T MRI scanner (Canon Medical Systems, Otawara, Tochigi, Japan). The MRI stroke protocol employed included axial DWI, axial Apparent Diffusion Coefficient (ADC); axial, sagittal, coronal T2 weighted (T2W); axial, coronal Fluid Attenuated Inversion Recovery (FLAIR); and axial, sagittal, coronal T1 weighted (T1W) sequences. Acute ischemic infarction on MRI appeared as a hyperintense area on T2W/FLAIR sequences and had a characteristic appearance of the hyperintense area showing diffusion restriction (DWI) with the corresponding hypointensity on its ADC map. It remains hypointense on TIW images.

The same consultant radiologist reported both the CT perfusion and MRI scans separately. Findings of CT perfusion and MRI stroke protocol imaging were recorded for detection of acute ischemic infarct by both modalities. Data collected were entered and analyzed using SPSS version 22 (IBM Inc., Armonk, New York). Mean with standard deviation was calculated for quantitative variables like age. Frequency and percentage were calculated for gender. Sensitivity, specificity, positive predictive value, and negative predictive value were calculated by a 2 x 2 table.

## Results

Of the 125 patients, 73 (58%) were male and 52 (42%) were female. The minimum age was 38 years, and the maximum age was 70 years with a mean + standard deviation of 56.12 ± 9.69 years.

**Figure 1:**
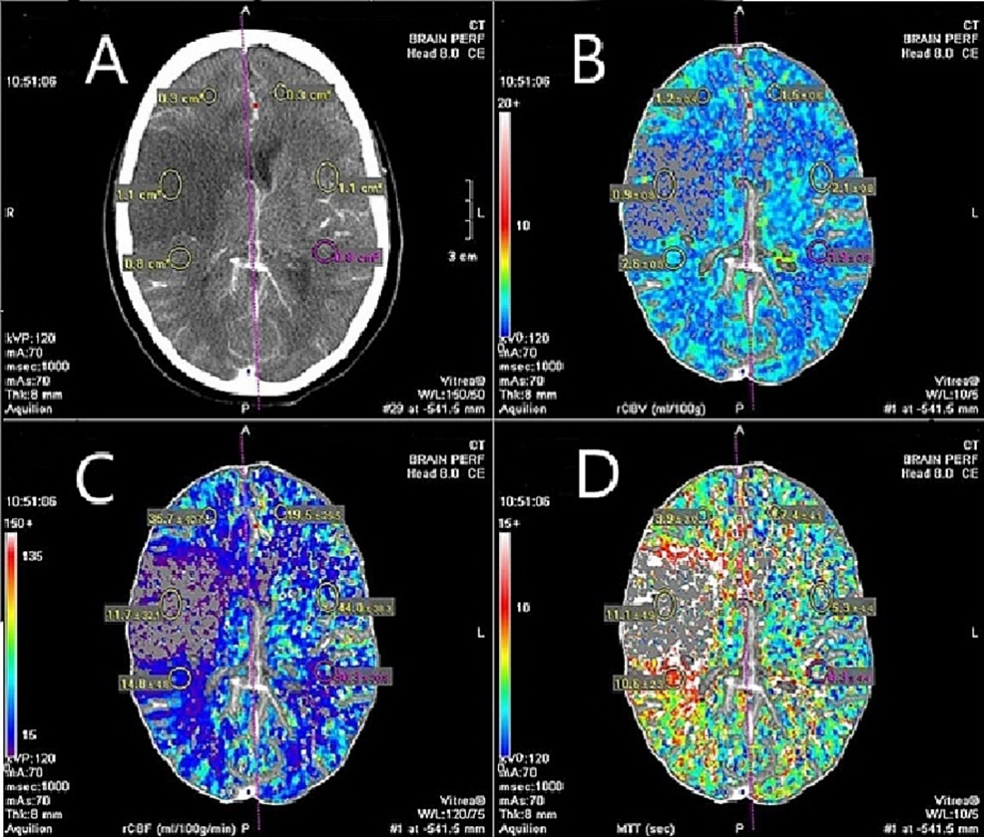
CT Perfusion images A. Non-contrast axial CT brain image showing right-sided acute ischemic infarction with regions of interest selected on the infarcted side and contralateral normal side. Assessment of perfusion parameters suggestive of acute ischemic infarction B. Decreased Cerebral Blood Volume. C. Decreased Cerebral Blood Flow. D. Increased Relative Mean Transit Time.

According to CT perfusion, acute ischemic infarction was detected in 86 (69%) patients while the infarction was not detected in 39 (31%) patients (Figure 1). According to MRI, acute ischemic infarction was detected in 120 (96%) patients and the infarction was not detected in 5 (4%) patients. The sensitivity of CT perfusion for detection of acute infarction was calculated as 70.83%, the specificity was 80%, the positive predicted value was 98.83, the negative predicted value was 10.25, and diagnostic accuracy was 71% (Table 1).

**Table 1:** 2x2 Table of CT Perfusion for detection of Acute Ischemic infarction [TP=True Positive, TN=True Negative, FP=False Positive, FN=False Negative]

CT Perfusion	MRI-DWI Findings		Total
	Acute Ischemic Infarct Detected	Acute Ischemic Infarct Not Detected	
Acute Ischemic Infarct Detected	TP= 85	FP= 1	86
Acute Ischemic Infarct Not Detected	FN= 35	TN= 4	39
Total	120	5	125

## Discussion

According to our study, CT perfusion provides a significantly accurate assessment of ischemic brain tissue, including both core and penumbra, with high predictive value.

CT perfusion is a strong diagnostic modality for accurate assessment of volumetric core/penumbra mismatch ratios, thus predicting the volume of potentially rescuable tissue at risk that can be saved from recanalization therapy [17,18]. It not only aids in the selection of stroke patients for reperfusion treatment but also has good prognostic value [19]. CT may not only help in the selection of candidates for endovascular intervention in conjunction with tPA therapy, but it can also help in those patients in whom the use of tPA is contraindicated [20,21].

CT perfusion is an attractive option to be adopted in health care setups, as it is a widely available, cost-effective, and time-saving technique; for urgent diagnosis and early intervention of in-need hyperacute stroke patients. The early reperfusion of blocked arteries significantly improves the life quality of patients [22]. Thus, CT perfusion not only decreases overall mortality and morbidity but also significantly reduces the economic and social burden of a country [23].

According to a meta-analysis conducted in 2015, pooled overall sensitivity for CT perfusion technique was 55.7%, whereas specificity was 92% [24]. Another study of 2017 concluded that sensitivity and specificity of CT perfusion scanning were 82% and 96%, respectively. This study highlighted the superiority of CT perfusion study over non-contrast CT (NCCT) brain explaining that although NCCT brain can detect ischemic infarct in most cases, it is of limited value in detecting the early acute ischemic infarction (within 24 hours of the onset of symptoms) and it is influenced by severity and size of the infarct. CT perfusion is superior as it can detect early signs of ischemic stroke not visible on non-contrast CT scan [25].

CT perfusion study can be used to detect the acute ischemic infarction in health care setups where MRI facility is not available, or MRI is out of order due to some reason. The use of CT perfusion study can be of prime importance in those circumstances where MRI is contraindicated, like in patients with pacemakers, cochlear implants, or any other metallic prosthesis and for patients with claustrophobia [26]. Another advantage of CT perfusion study is the reduced time requirement as compared to MRI scanning; hence it can be employed as an emergency procedure in critically ill patients needing prompt intervention [27].

The disadvantages of CT perfusion studies include exposure to ionizing radiation, the use of iodinated contrast, and the inability of scanners to image the entire brain for perfusion imaging in many healthcare facilities, including our setup.

## Conclusions

CT perfusion imaging provides high predictive value in the assessment of acute ischemic infarct in stroke patients. This widely available and time-effective modality aids in the triage of patients for immediate endovascular intervention leading to maximal neurological benefit and improving outcomes. This technique can be used as an alternative in cases of non-availability or contraindication of MRI.
